# Gender Differences Among Urologists in the Assessment and Utilization of ESWL Treatment: Insights from a Multinational Survey of 3747 Participants

**DOI:** 10.3390/jcm15187306

**Published:** 2026-09-20

**Authors:** Hajira Karim, Abdullah Hamdullah Azeemi, Duha Yahya, Mona Ayran, Abdallah Mohammad Ibrahim Abu Dayah, Rahaf Salaam, Emad Sibai, Imadeddine Boudjatit, Kani Barzng, Youssef Shalaby, Mehmet Kocak, Guohua Zeng, Valentin Pavlov, M. Pilar Laguna, Jean de la Rosette

**Affiliations:** 1 International School of Medicine, Istanbul Medipol University, 34810 Istanbul, Türkiye; hajira.karim@std.medipol.edu.tr (H.K.); azeemi.1981@gmail.com (A.H.A.); dohaosamayehia@gmail.com (D.Y.); monaayran@gmail.com (M.A.); abdullahabudayyeh9@gmail.com (A.M.I.A.D.); rahaf.salaam@gmail.com (R.S.); sibaiemad10@gmail.com (E.S.); kani.hasseb@gmail.com (K.B.); mehmetkocak@medipol.edu.tr (M.K.); m.p.lagunapes@gmail.com (M.P.L.); 2 Research Institute for Health Sciences and Technologies (SABITA), Istanbul Medipol University, 34810 Istanbul, Türkiye; 3 Başakşehir Çam ve Sakura City Hospital, 34480 Istanbul, Türkiye; 4 International School of Medicine, Bahçelievler Medical Park Hospital, Altinbas University, 34147 Istanbul, Türkiye; iboudjatit2003@gmail.com (I.B.); youssefabdelsalam.25@gmail.com (Y.S.); 5 Multi-Omics Design and Analysis Studio (MODAS-SABITA), Istanbul Medipol University, 34810 Istanbul, Türkiye; 6 Department of Urology and Guangdong Key Laboratory of Urology, The First Affiliated Hospital of Guangzhou Medical University, Guangzhou 510230, China; gzgyzgh@vip.sina.com; 7 Department of Urology and Oncology, Bashkir State Medical University, 450008 Ufa, Russia; pavlov@bashgmu.ru; 8 Department of Urology, Istanbul Medipol Mega University Hospital, 34214 Istanbul, Türkiye

**Keywords:** ESWL, gender, urinary stones, multinational survey, practice patterns, training

## Abstract

**Background/Objectives**: This study aimed to examine gender-associated differences in extracorporeal shock-wave lithotripsy (ESWL) utilization as well as urolithiasis diagnostic evaluation, treatment strategy, and follow-up across a multinational sample of urologists and trainees. **Methods**: A multinational cross-sectional survey of members of the Société Internationale d’Urologie (SIU) was conducted from June to July 2022 in seven languages. The survey was completed by 3747 urologists and trainees (283 female and 3464 male respondents) from 108 countries. To reduce baseline differences, propensity-score matching was performed with exact matching on years of clinical practice, continent, and career stage, yielding a matched cohort of 1191 respondents (279 female and 912 male). Categorical comparisons used Cochran–Mantel–Haenszel analyses stratified by a matched-set identifier, and 0–10 scored items were compared using stratified Wilcoxon rank-sum analyses with the matched set as the stratum. **Results**: In the matched cohort, annual procedural-volume distributions differed by gender for ureterorenoscopy (URS) (*p* = 0.0009), open surgery (*p* = 0.002), laparoscopy/robotics (*p* = 0.010), and percutaneous nephrolithotomy (PCNL) (*p* = 0.018), whereas ESWL volume did not differ significantly (*p* = 0.077). Among ESWL-specific categorical practices, coupling-gel use differed by gender (*p* = 0.029), while alpha-blocker use, antibiotic prophylaxis, JJ-stent use, stone-fragment collection, ESWL-machine type, and operator did not. Among the 851 respondents that completed the 0–10 practice items (186 female, 665 male), urine-culture assessment (8.4 vs. 7.6; *p* = 0.030), complete blood count (8.0 vs. 7.5; *p* = 0.047), and repeating ESWL within 7 days (2.5 vs. 3.4; *p* = 0.048) showed nominal gender-associated differences. The majority of other scored items were similar, including computed tomography (CT) urography (*p* = 0.055). Because these analyses were exploratory and no multiplicity adjustment was applied, nominally significant findings should be interpreted as hypothesis-generating. **Conclusions**: In this multinational survey of SIU members, most self-reported ESWL-specific practices were similar between female and male respondents after matching on key demographic, geographic, and professional characteristics. Gender-associated differences were observed in procedural-volume distributions as well as a limited number of reported assessments and procedural items. These findings are exploratory associations rather than causal effects of gender and may reflect measured and unmeasured professional, institutional, geographic, and training-related factors.

## 1. Introduction

Urolithiasis, or urinary stones, is a highly prevalent condition, with a lifetime risk approaching 15% and recurrence rates nearing 50% within ten years of initial diagnosis [1]. This condition and its associated sequelae represent a persistent challenge for patients and healthcare systems worldwide. Alongside conservative measures, contemporary urinary stone management relies primarily on three procedural modalities: extracorporeal shock-wave lithotripsy (ESWL), ureterorenoscopy (URS), and percutaneous nephrolithotomy (PCNL) [1].

Since the first successful clinical treatment of a kidney stone using ESWL in 1980, the technique has profoundly transformed urinary stone management. Within five years of its introduction, ESWL became one of the most commonly performed interventions for renal and ureteral calculi, offering a completely non-invasive alternative to URS and PCNL [2,3,4]. Early experience with the Dornier HM3 reported success rates exceeding 90%; however, subsequent studies demonstrated considerable variability in treatment outcomes, even when the same lithotripter was used [2,3,4].

Over subsequent decades, however, the role of ESWL has waned because its clinical success depends on multiple stone- and patient-related factors, including stone size and density, renal anatomy, skin-to-stone distance, body habitus, and renal function as well as machine-related variables such as shock rate, energy settings, focusing accuracy, and coupling quality [2]. Newer-generation lithotripters have not consistently reproduced the excellent outcomes of early devices, and comparative data, summarized in contemporary guidelines, show superior stone-free rates for URS in many ureteric scenarios and for PCNL in larger-sized renal stones [1,2]. Consequently, daily practice has shifted towards URS and PCNL, and the indications for ESWL have narrowed, even though it remains a recommended option in major international guidelines for appropriately selected urinary stones [1].

Beyond technology and stone characteristics, ESWL utilization varies by physician and system-level factors. International data suggest substantial variation in access to lithotripters, training exposure, case volume, and delegation of treatments to non-physicians. The decision to offer ESWL often appears to depend not only on high-level evidence but also on individual preference, local expertise, device availability, and economic considerations [2,3]. As studied in other fields, the gender of the treating physician is a potential contributor to the variability of treatment decision-making and patient outcomes in clinical practice [5,6,7,8].

Urology remains a predominantly male specialty. Across medicine, a growing body of evidence in the literature suggests that physician gender may be associated with communication, tolerance of uncertainty, risk perception, adherence to guidelines, and patterns of test ordering or procedure use [5,6,7,8]. In surgical disciplines, some observational studies have reported differences in patient outcomes according to surgeon gender after adjustment for measured case-mix and institutional factors [7,9]. In urology, gender differences have been described in subspeciality choices and procedural focus, alongside unequal access to mentorship, leadership, and some high-technology practice environments. These observations provide a rationale for examining whether reported ESWL practice patterns differ by gender.

ESWL provides a useful setting in which to examine such variation because its use involves multiple clinical decision points, including pre-treatment evaluation and patient selection, retreatment timing, adjunctive measures, and post-treatment imaging [1,2,3]. However, systematic evidence on whether these self-reported ESWL practice patterns differ by the gender of the treating urologist remains limited.

The primary objective of this study was to characterize gender-associated differences across major domains of reported ESWL practice in a multinational sample of urologists and trainees. These domains included procedural utilization and case volume; pre-treatment clinical, laboratory, and imaging assessment; perceptions of contraindications and special clinical scenarios, peri-procedural and adjunctive management; number and timing of treatment sessions; and post-treatment imaging for stone-free assessment. The study was designed to describe associations and practice variation rather than to infer a causal effect of physician gender.

## 2. Materials and Methods

The ESWL survey was conducted from June to July 2022 as an anonymous, voluntary online survey of members of the Société Internationale d’Urologie (SIU). A total of 3747 urologists and trainees from 108 countries across five continents completed the survey. The study was conducted in accordance with the Declaration of Helsinki. The objectives of the survey and intended use of the data were described in the invitation, and participants provided consent through voluntary completion of the survey. No personally identifiable information or participant-level data were collected. The present secondary analysis of the anonymized survey data was approved by the Istanbul Medipol University Non-Interventional Clinical Research Ethics Committee (approval number E-10840098-50.04-3324). Because participation was voluntary and was drawn from the SIU survey network, the sample should be considered multinational rather than representative of the global urologist population.

**Figure 1:**
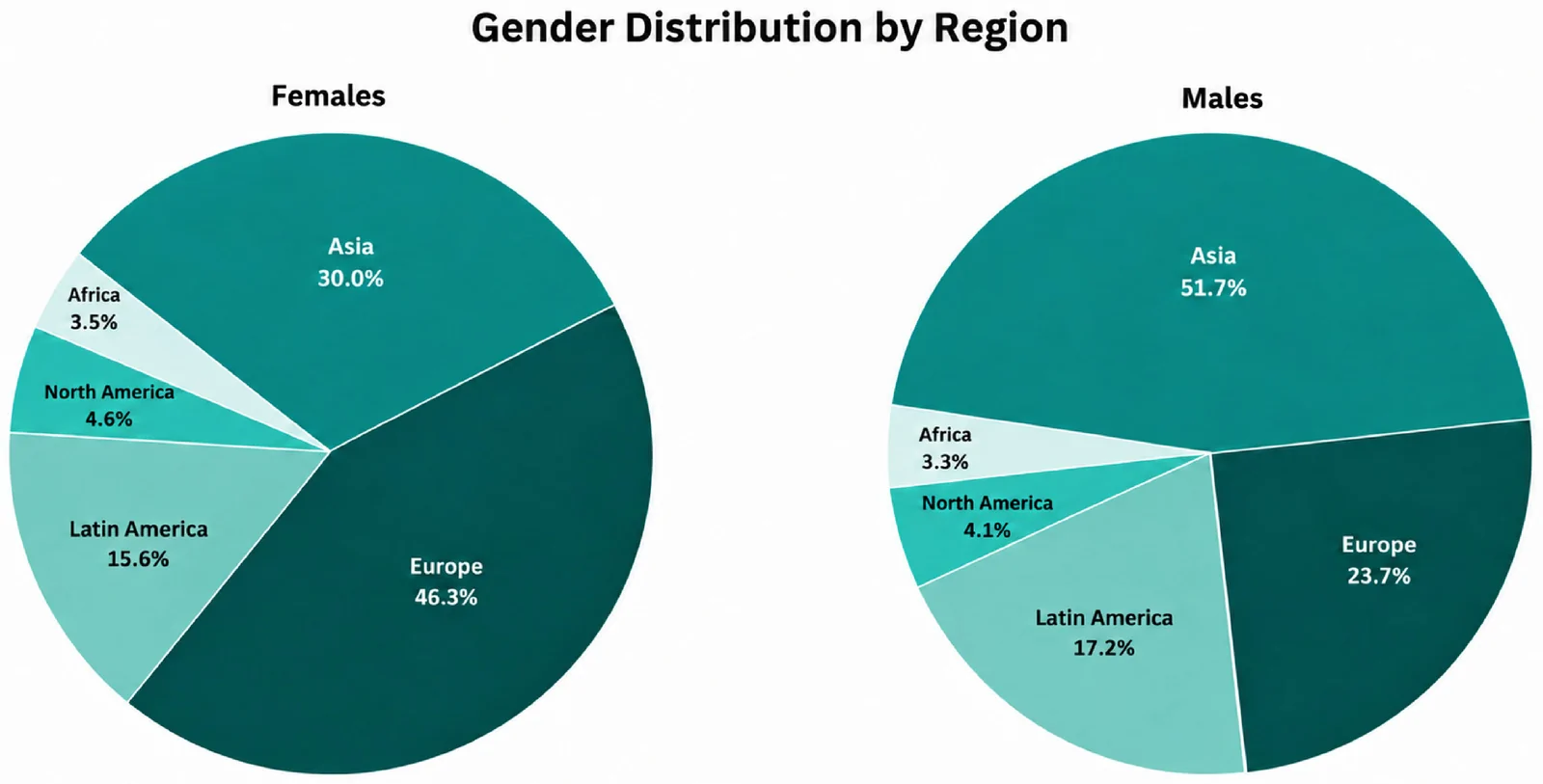
Continent distribution of the full (unmatched) survey population by gender (*n* = 3747), showing the significant geographic imbalance (*p* < 0.0001) that motivated exact matching by continent.

Female urologists represent a small fraction of urologists (7.5% of the full survey sample) and differed from male respondents in several baseline professional and demographic characteristics, including age, geographic distribution, years of clinical practice, and career stage. The geographic imbalance was particularly evident in the unmatched cohort (Figure 1), motivating the use of a matching strategy to improve comparability between gender groups.

**Figure 2:**
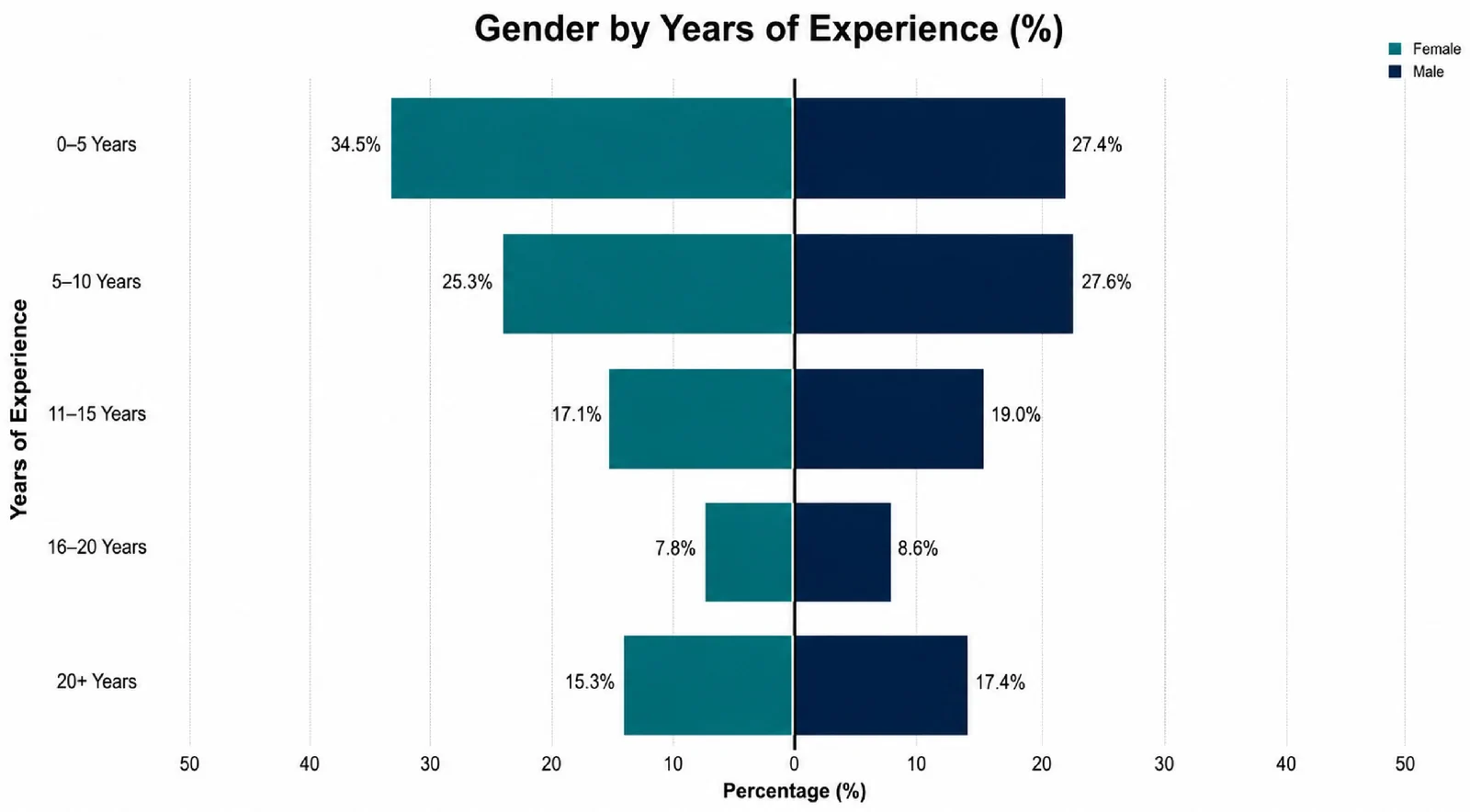
Distribution of years of clinical practice in the full (unmatched) survey population by gender (*n* = 3747), showing the earlier-career skew among female respondents; this variable was used as an exact-matching factor.

A similar early-career skew was observed in the distribution of years in clinical practice among female respondents (Figure 2).

To improve comparability between female and male respondents, propensity-score matching was performed using the SAS PROC PSMATCH procedure (SAS 9.4, SAS Institute, Cary, NC, USA). Female respondents were designated as the treated group and male respondents as the control group solely for purposes of the matching algorithm. Propensity scores were estimated using a main-effects logistic regression model including age as a continuous variable and years of clinical practice, career stage, and continent as categorical variables. Greedy nearest-neighbor matching without replacement was performed on the logit of the propensity score, requesting up to four male controls for each female respondent and processing female respondents in descending order of propensity score. Years of clinical practice, continent, and career stage were additionally required to match exactly. A caliper width equal to 0.10 pooled standard deviations of the logit propensity score was imposed; thus, the value 0.10 did not represent a difference in age or in the raw propensity score. Because the exact-matching and caliper restrictions could prevent four eligible controls from being identified for every female respondent, the realized matching ratio could be smaller than 1:4. The matching procedure resulted in 279 female respondents matched with 912 male respondents (*n* = 1191; average realized ratio of approximately 1:3.27). Matching was performed without replacement. No imputation was required because the variables used for matching had no missing values.

**Figure 3:**
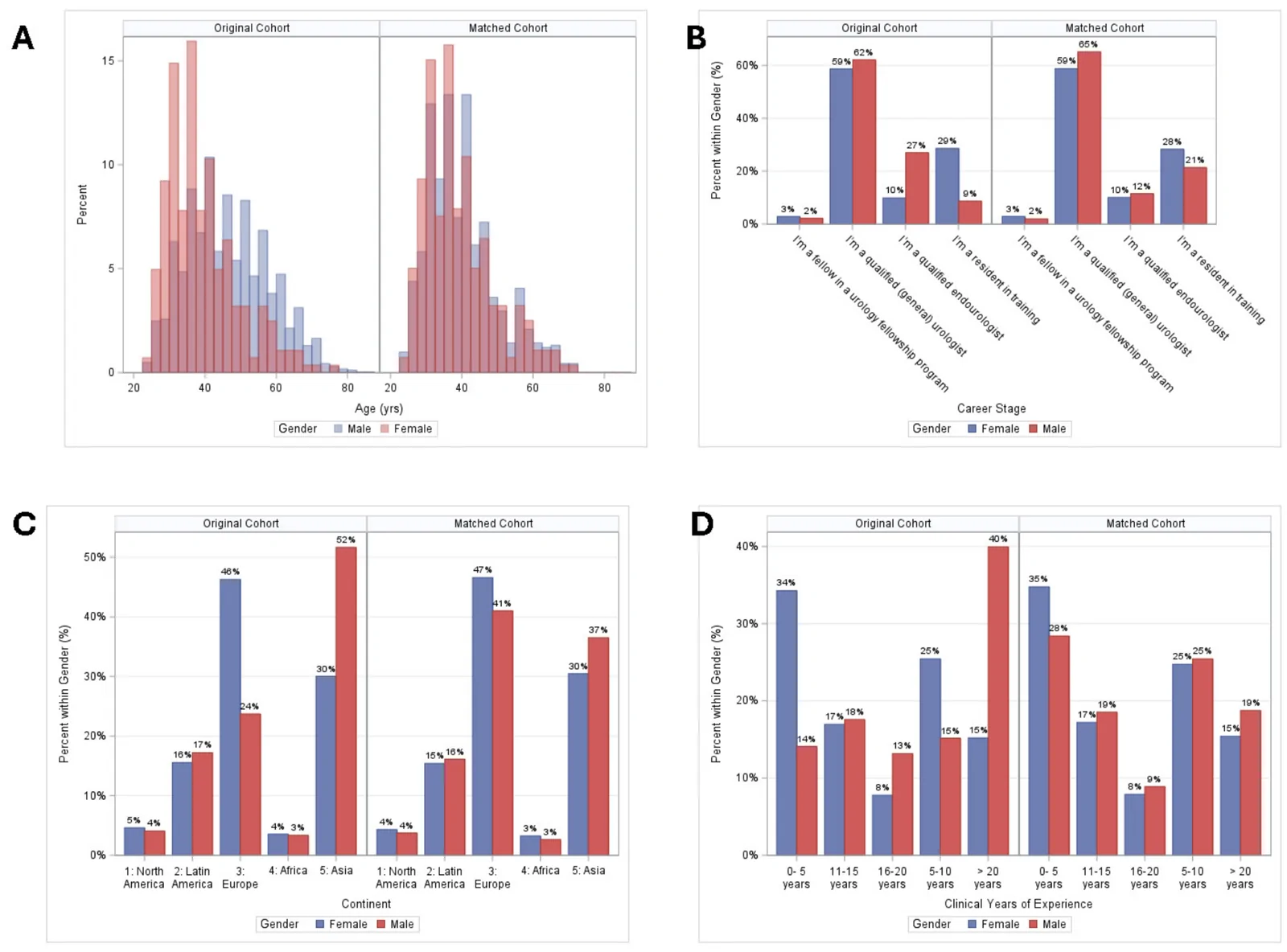
Gender-specific distributions of the variables used in propensity-score matching: (**A**) age distribution in the original and matched cohorts; (**B**) career-stage distribution in the original and matched cohorts; (**C**) continent distribution in the original and matched cohorts; (**D**) years of clinical practice distribution in the original and matched cohorts.

Propensity-score matching was used as a design-stage procedure to construct female and male respondent cohorts with improved comparability in demographic, geographic, and professional characteristics rather than to estimate a causal treatment effect. Covariate balance before and after matching was assessed using standardized mean differences and visual inspection of the matching diagnostics; the improvement in the distributions of key matching characteristics is illustrated in Figure 3. An absolute standardized mean difference below 0.10 was considered indicative of negligible residual imbalance. Career stage differed substantially by gender in the unmatched cohort and was therefore added as an exact-matching criterion. Years of clinical practice and continent were also exact-matching variables, whereas age contributed to the propensity-score model. Post-matching balance diagnostics demonstrated substantial improvement in comparability between gender groups; the pre- and post-matching distributions of key characteristics are shown in Figure 3.

The matching for residents and fellows was conducted as a separate analysis cohort. In this cohort, 79 female urologists at the residency and fellowship stage were matched with 195 male counterparts. Because the survey included both practicing urologists and trainees, results were interpreted in the overall matched cohort and, separately, in the resident/fellow subgroup to reduce the influence of career stage when assessing gender-associated practice differences.

The principal categorical comparisons were based on the matched cohort of 1191 respondents (279 female and 912 male). Scored ESWL practice-pattern items were available for 851 respondents (186 female and 665 male). Of the 340 matched respondents without scored practice-item data, 296 (87.1%) either did not have an ESWL device at their hospital or reported an ESWL procedural volume of zero, indicating that most missing practice-item responses reflected non-applicability of the survey branch rather than generalized item nonresponse. The 0–10 items represented ratings of perceived importance or level of agreement, depending on the survey item, with higher values (i.e., closer or equal to 10) indicating greater importance or agreement. These individual items were survey-specific and were not interpreted as a validated composite psychometric scale.

Descriptive counts, percentages, means, standard deviations, medians, and interquartile ranges were calculated from the observed matched cohort without propensity-score outcome weighting. Categorical outcomes were compared using Cochran–Mantel–Haenszel analyses stratified by the matched-set identifier generated by PROC PSMATCH. Scored 0–10 items were compared using stratified Wilcoxon rank-sum analyses with the matched-set identifier as the stratum. All tests were two-sided with a nominal significance level of 0.05. Because this secondary analysis evaluated multiple practice-pattern items without a prespecified primary outcome, the analyses were considered exploratory and no adjustment for multiple testing was applied. All analyses were conducted using SAS version 9.4 (SAS Institute, Cary, NC, USA).

Generative AI was not used in the design, conduct, analysis, or interpretation of this study.

## 3. Results

A total of 3747 urologists and trainees completed the survey, comprising 283 female (7.55%) and 3464 male (92.45%) respondents. Because female respondents differed at baseline (Figure 1 and Figure 2), the primary gender comparisons were performed in the propensity-matched cohort of 1191 respondents (279 female, 912 male); the resident/fellow subgroup (79 female, 195 male) was analyzed separately. Respondents were represented across all major geographic regions, with the largest proportions originating from Asia, Europe, and Latin America. Because participation was voluntary and SIU-based, these results describe a large multinational respondent sample rather than a population-representative global cohort.

**Figure 4:**
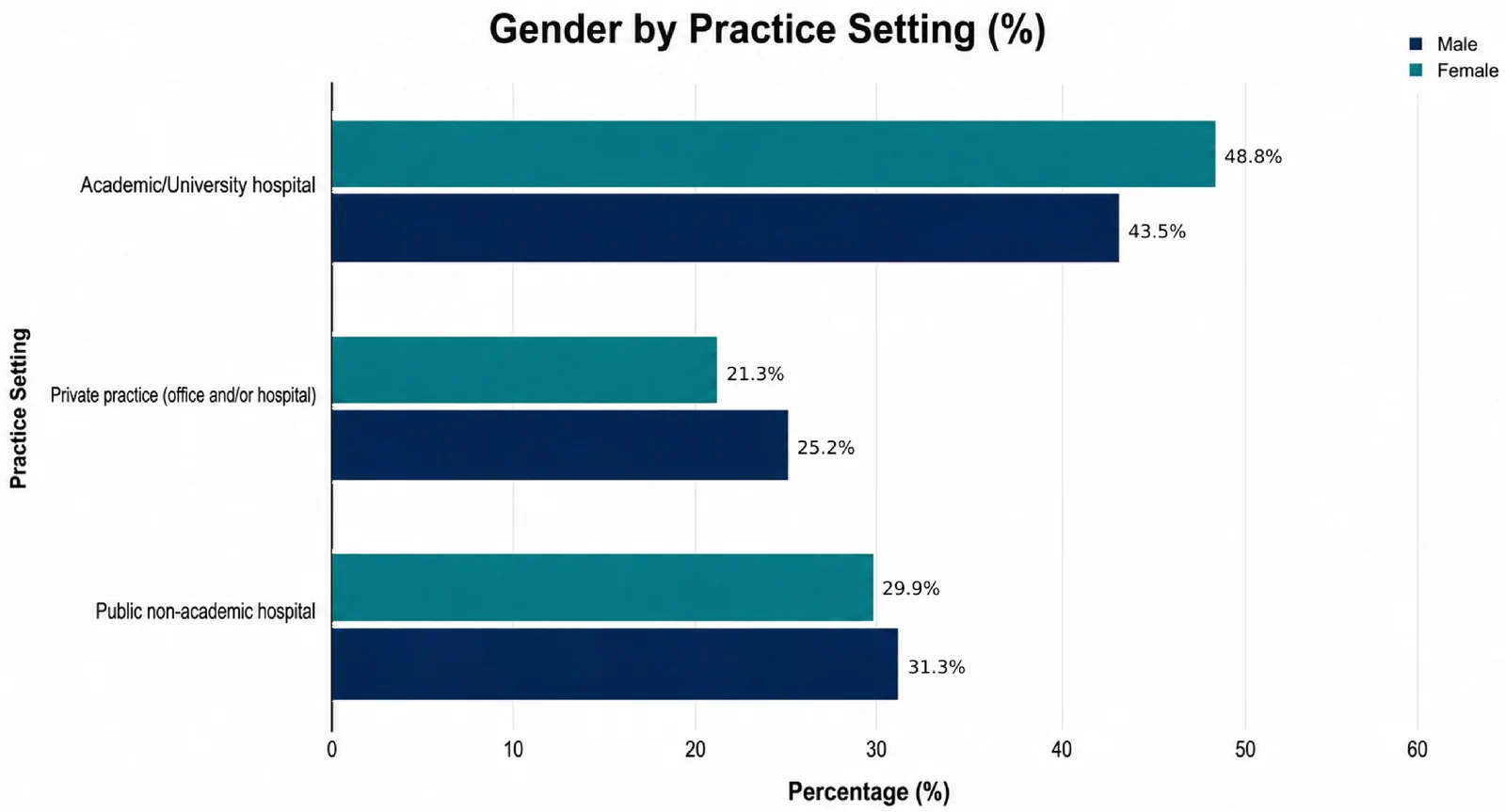
Gender distribution across practice settings.

Practice setting was not used as an exact-matching criterion and was examined descriptively as an additional professional characteristic, showing no significant difference by gender (*p* = 0.22, Figure 4).

**Figure 5:**
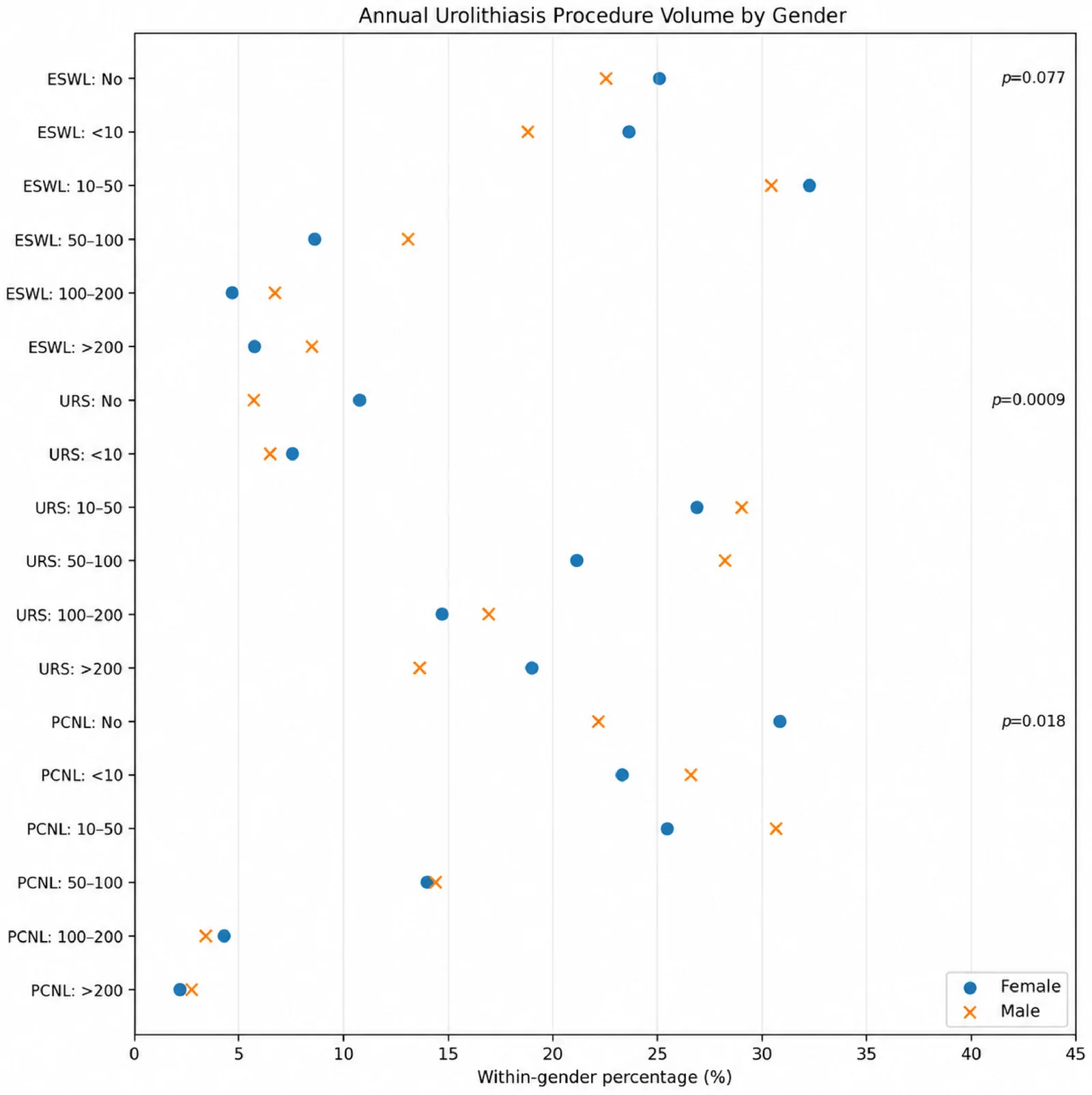
Gender-stratified distributions of annual procedural-volume categories for ESWL, URS, and PCNL in the matched cohort. Percentages were calculated within gender among respondents with non-missing procedure-volume data. Inferential *p*-values were obtained from Cochran–Mantel–Haenszel analyses stratified by matched-set identifier. Most categorical ESWL-specific practice patterns were similar between female and male respondents in the matched cohort. Stone-fragment collection after ESWL did not differ by gender (*p* = 0.55), nor did the personnel responsible for performing ESWL (*p* = 0.83), ESWL-machine type (*p* = 0.33), access to ESWL service (*p* = 0.42), or antibiotic prophylaxis use (*p* = 0.098). Coupling-gel use was the only categorical ESWL-specific practice item showing a nominal gender-associated difference (*p* = 0.029): 69.9% of female respondents and 78.9% of male respondents reported using coupling gel. Alpha-blocker use (63.4% vs. 73.0%; *p* = 0.085) and JJ-stent use (28.5% vs. 21.4%; *p* = 0.12) did not reach nominal statistical significance after accounting for the matched set (Figure 6).

Annual procedural volume showed procedure-specific gender patterns. ESWL case-volume distribution did not differ significantly between female and male respondents (*p* = 0.077). Both groups were most commonly represented in the 10–50 cases/year category. Although female respondents were numerically more represented in lower-volume ESWL categories, including no cases and <10 cases/year, and male respondents were more represented in higher-volume categories, this difference did not reach statistical significance. In contrast, URS case-volume distribution differed by gender (*p* = 0.0009). Open-surgery volume (*p* = 0.002) and laparoscopy/robotics volume (*p* = 0.010) also differed between gender groups in the matched-set-stratified analyses. PCNL case-volume distribution also differed between female and male respondents (*p* = 0.018), whereas ESWL volume did not. These nominal associations indicate that procedural-volume differences persisted for several urolithiasis procedures even after matching on age, geography, clinical experience, and career stage; given the exploratory nature of the analysis and the number of comparisons, these nominal associations should be interpreted cautiously (Figure 5).

**Figure 6:**
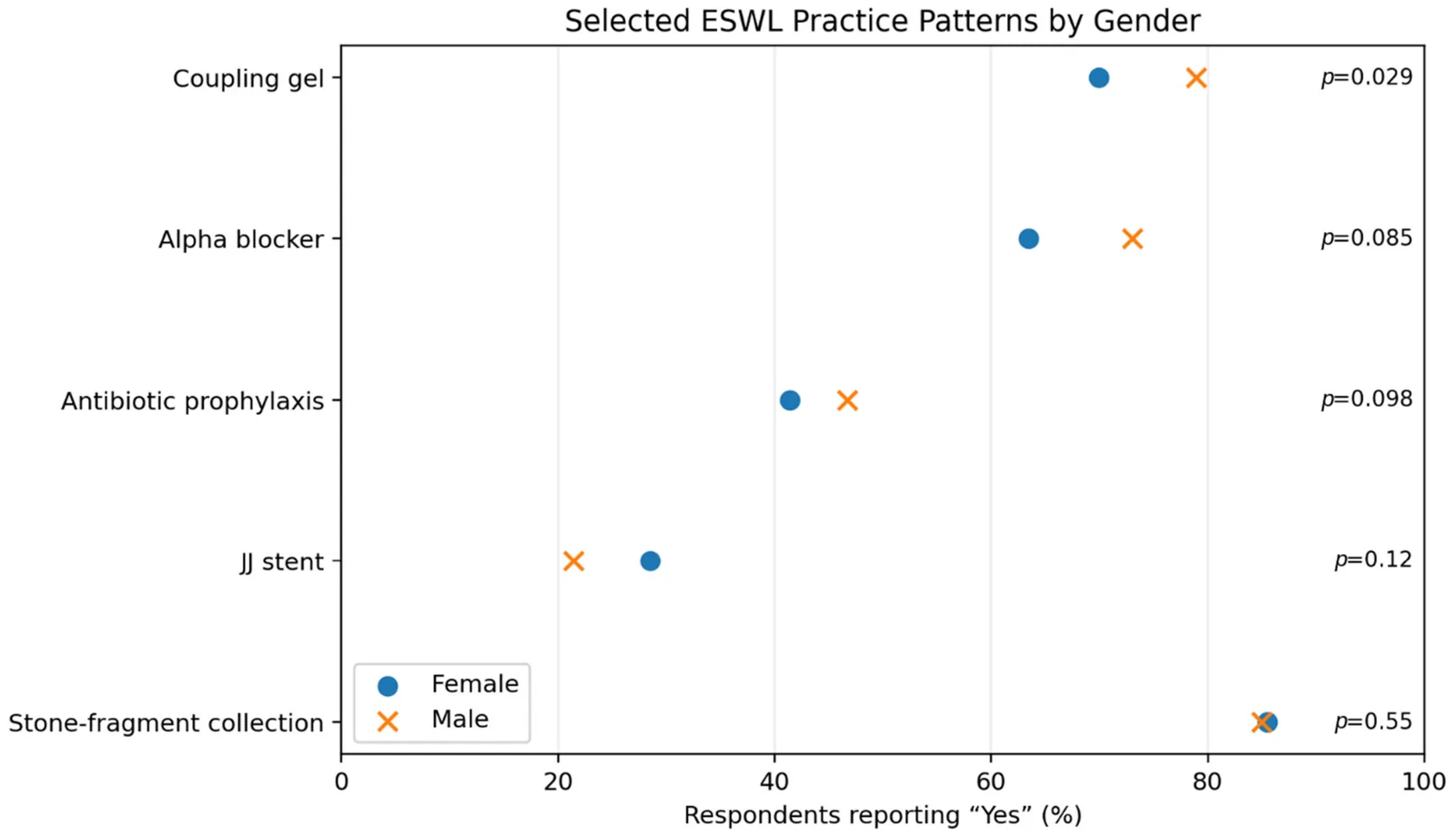
Selected categorical ESWL practice patterns by gender in the matched cohort. Points represent the within-gender percentage of respondents reporting “Yes” for each item; *p*-values were obtained from Cochran–Mantel–Haenszel analyses stratified by matched-set identifier. Only coupling-gel use reached the nominal 0.05 significance threshold; the remaining categorical practice items displayed did not.

**Figure 7:**
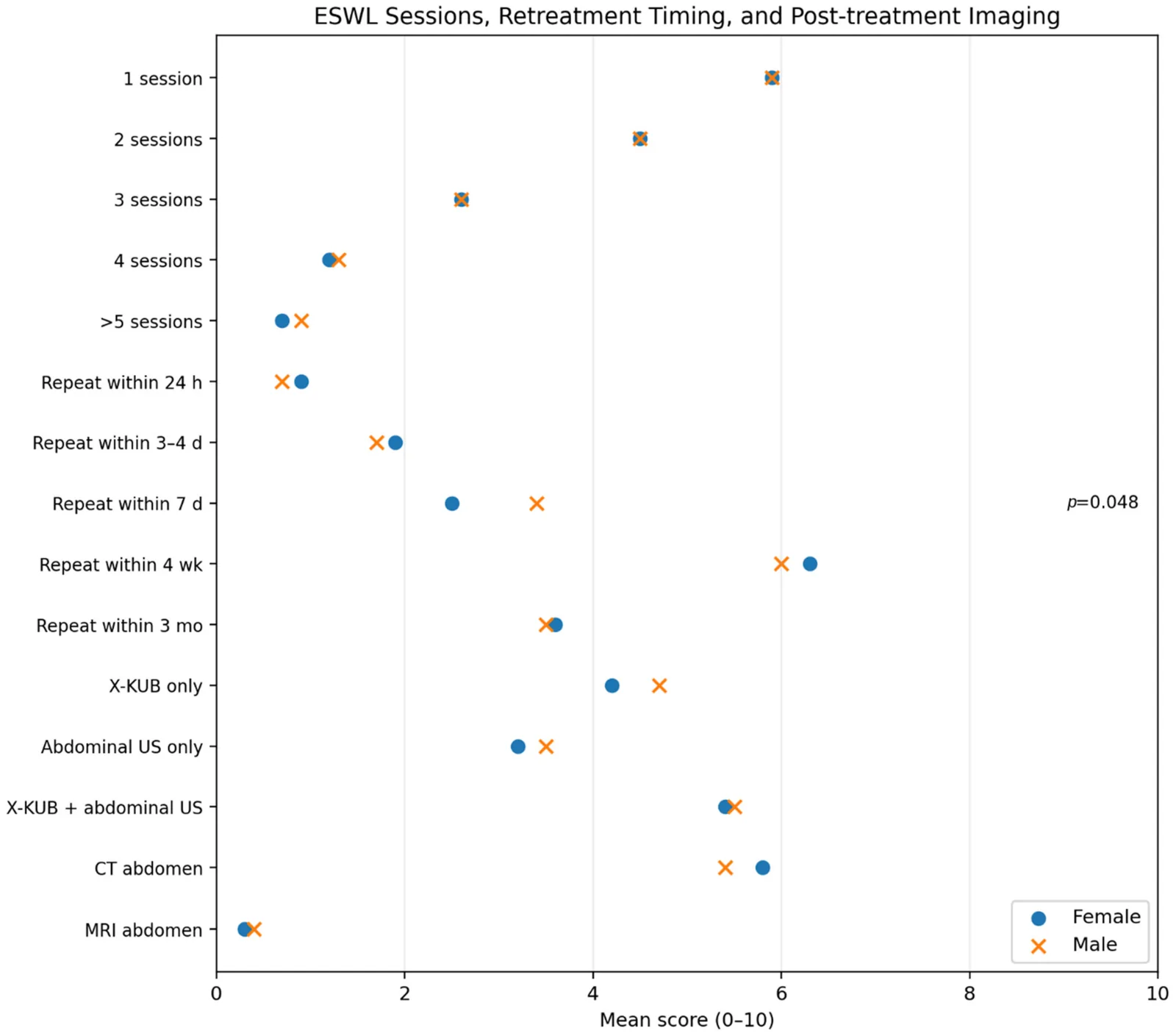
Mean 0–10 scores for ESWL session patterns, repeat-treatment timing, and post-treatment imaging evaluation by gender in the scored-item subgroup. Points represent group means for female and male respondents. The *p*-value is shown for the only item reaching nominal statistical significance in the matched-set-stratified Wilcoxon analysis. A nominal difference was observed only for repeating ESWL within 7 days (*p* = 0.048), with a higher mean score among male respondents. Other session and post-treatment imaging items were not statistically significant.

ESWL session patterns were broadly similar between female and male respondents. Among the repeat-treatment interval items, the score for repeating ESWL within 7 days was higher among male respondents than female respondents (mean 3.4 vs. 2.5; *p* = 0.048). Scores for repeat treatment within 24 h, within 3–4 days, within 4 weeks, and within 3 months did not differ significantly between gender groups. Because *p* = 0.048 was nominal and multiple items were evaluated, this finding is considered hypothesis-generating (Figure 7).

Post-ESWL imaging evaluation was similar between female and male respondents. None of the evaluated follow-up imaging modalities reached nominal statistical significance in the matched-set-stratified analyses, including kidneys, ureters, and bladder X-ray (X-KUB)-only assessment (*p* = 0.069), computed tomography (CT) of the abdomen, abdominal ultrasound, combined X-KUB and abdominal ultrasound, and magnetic resonance imaging (MRI) of the abdomen (Figure 7).

Pre-ESWL clinical assessment scores were generally similar between female and male respondents. Clinical history and abdominal physical examination did not differ significantly in the matched-set-stratified analyses.

Within the laboratory domain, urine-culture assessment had a higher mean score among female respondents than male respondents (8.4 vs. 7.6; *p* = 0.030), as did complete-blood-count assessment (8.0 vs. 7.5; *p* = 0.047). Urinalysis and coagulation-testing scores did not differ significantly. These *p*-values are nominal and should be interpreted within the exploratory framework of the study.

**Figure 8:**
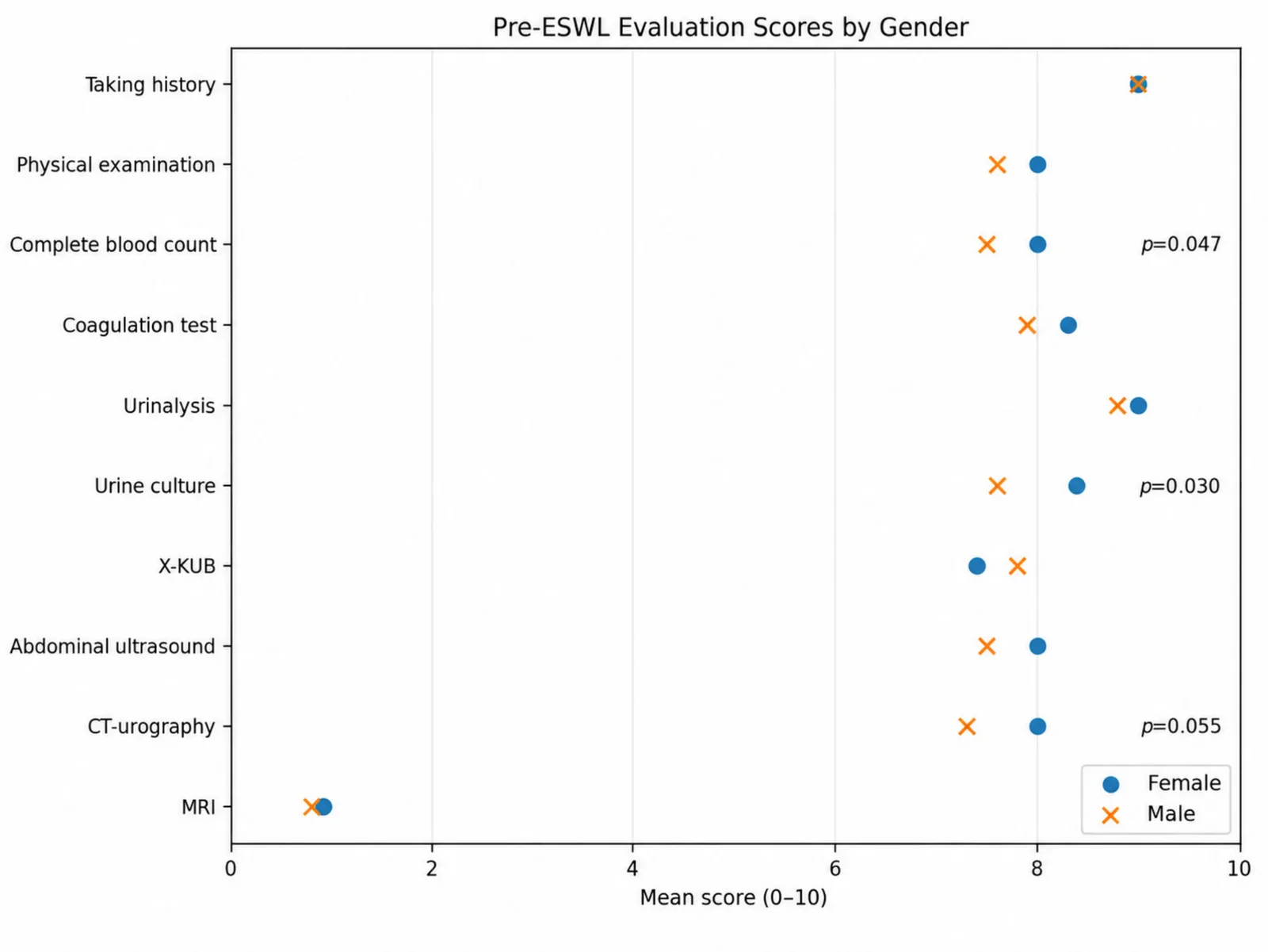
Mean 0–10 scores for pre-ESWL clinical, laboratory, and imaging evaluation items by gender in the scored-item subgroup. Points represent group means for female and male respondents. *p*-values are displayed for urine culture, complete blood count, and CT urography, the items with *p* ≤ 0.055 in the matched-set-stratified Wilcoxon analyses. Nominal differences were observed for urine-culture assessment (*p* = 0.030) and complete blood count (*p* = 0.047). CT urography was borderline but did not reach the nominal 0.05 threshold (*p* = 0.055).

No item reached nominal statistical significance within the imaging domain. CT-urography showed the greatest between-gender difference (mean 8.0 among female respondents vs. 7.3 among male respondents; *p* = 0.055), whereas abdominal ultrasound (8.0 vs. 7.5; *p* = 0.15), X-KUB, and MRI were all statistically similar (Figure 8).

Assessment of ESWL-related contraindications and special clinical scenarios showed little gender-associated variation in the analysis. Pregnancy, anticoagulation medication, morbid obesity, and the other evaluated clinical scenarios did not differ significantly between female and male respondents.

No infection-related or stone-composition item reached nominal statistical significance. In particular, the radiolucent-stone score was higher numerically among female respondents (5.8 vs. 5.2) but was not statistically different after accounting for the matched set (*p* = 0.39). Prevalence of recurrent urinary tract infection and struvite stones was likewise similar between gender groups.

**Figure 9:**
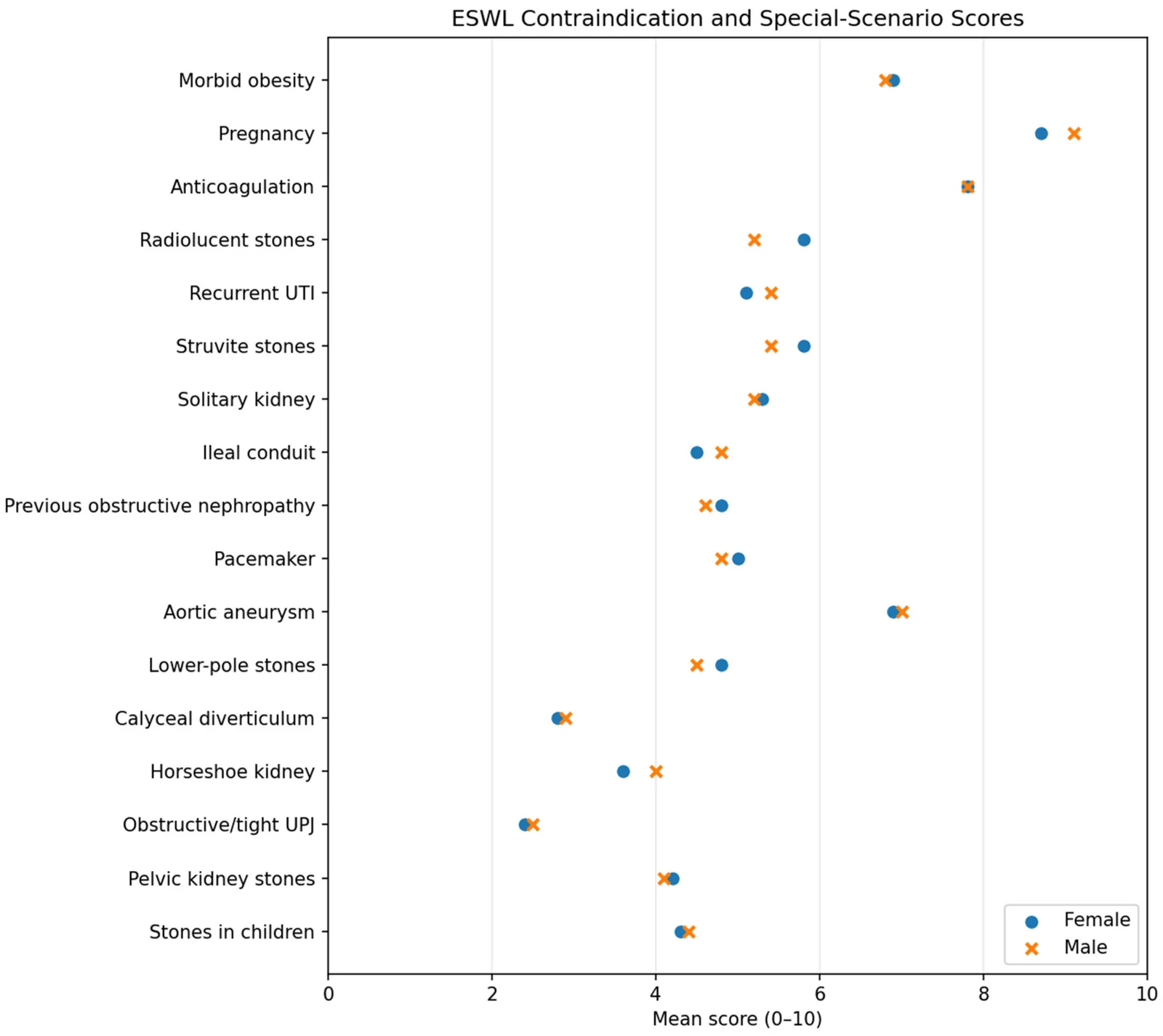
Mean 0–10 scores for ESWL-related contraindications and special clinical scenarios by gender in the scored-item subgroup. Points represent group means for female and male respondents; none of the displayed items reached the nominal 0.05 significance threshold in matched-set-stratified Wilcoxon analyses. No contraindication or special-clinical-scenario score reached the nominal 0.05 significance threshold in the matched-set-stratified analyses.

Anatomical and complex urinary tract scenarios were also comparable between female and male respondents. No nominally significant gender-associated differences were observed for solitary kidneys, ileal conduits, previous obstructive nephropathy, pacemakers, aortic aneurysms, lower-pole stones, calyceal diverticulum stones, horseshoe kidneys, obstructive or tight ureteropelvic junctions, pelvic kidney stones, or stones in children (Figure 9).

Overall, the propensity-score matching strategy substantially improved comparability between female and male respondents in the baseline characteristics targeted by the matching algorithm. In the matched analysis, most ESWL-specific practices were similar between gender groups. Nominal differences were limited to coupling-gel use among categorical ESWL practice items and to urine-culture assessment, complete blood count, and repeat ESWL within 7 days among the scored items. Procedural-volume distributions differed for several urolithiasis procedures, including URS, open surgery, laparoscopy/robotics, and PCNL. All findings should be interpreted as exploratory because multiple comparisons were conducted without multiplicity adjustment.

## 4. Discussion

This secondary analysis of a multinational ESWL survey of 3747 SIU respondents examined whether self-reported ESWL practice patterns differed by respondent gender after constructing demographically, geographically, and professionally comparable matched cohorts. The matched-set-adjusted analyses showed that most ESWL-specific practices were similar between female and male respondents. Nominal differences were observed for coupling-gel use, urine-culture assessment, complete blood count, and repeat ESWL within 7 days, while procedural-volume distributions differed for several urolithiasis procedures. These findings represent exploratory associations in self-reported survey data and should not be interpreted as causal effects of physician gender or as direct measures of clinical competence, risk preference, or quality of care.

### 4.1. Gender Representation and Structural Context

The survey confirms a substantial gender disparity in global urology: 92.4% of respondents were male and only 7.55% were female [10]. Female respondents were disproportionately concentrated in Europe and Asia and were more commonly represented in training or early professional stages, while male respondents were more frequently represented in qualified endourologist roles [11,12]. These baseline differences could plausibly influence procedural exposure and reported practice patterns. For this reason, career stage was added as an exact-matching variable, along with years of clinical practice and continent. Residual differences in unmeasured training opportunities, institutional resources, and procedural exposure may nevertheless remain.

The geographic distribution of female respondents should also be considered when interpreting these findings. Female representation differed across regions, with higher proportions in Europe and Asia and lower representation elsewhere. This uneven distribution may reflect regional differences in access to urology training, mentorship, workforce structure, and opportunities for women in surgical specialties. Therefore, some observed gender-associated practice patterns may partly reflect geographic and health-system variation rather than gender alone.

The present survey did not use a prespecified guideline-concordance score, and individual practices cannot be classified as appropriate or inappropriate without patient-level clinical context. Accordingly, this study does not characterize either gender as more guideline-concordant. Instead, the findings are described neutrally as differences in reported practice patterns, with current guideline recommendations cited only when discussing specific practices for which the clinical context permits a meaningful comparison [13,14].

### 4.2. Pre-Treatment Assessment: Gender-Associated Differences in Reported Evaluation Practices

Pre-ESWL assessment practices were broadly similar between female and male respondents. In the matched-set-stratified analysis, urine-culture and complete blood count scores showed nominal differences, whereas CT urography was borderline (*p* = 0.055) and abdominal ultrasound was not statistically different. These findings indicate limited variation in self-reported assessment practices but do not establish that one group performs a more comprehensive, cautious, or clinically appropriate evaluation. The appropriateness of individual laboratory and imaging tests depends on clinical context and patient characteristics [1,2,3,13,14].

### 4.3. Reported Retreatment and Interval Planning

ESWL session patterns were broadly similar between female and male respondents. The score for repeating ESWL within 7 days showed a nominal gender-associated difference (*p* = 0.048), whereas the other evaluated retreatment intervals did not. Retreatment timing may be influenced by center protocols, case mix, stone characteristics, procedural volume, and local practice patterns. Because these factors and individual risk preferences were not directly measured, this association should not be interpreted as evidence that one gender is more or less risk-tolerant.

### 4.4. Adjunctive Medications

Alpha-blocker use and antibiotic prophylaxis did not differ significantly between female and male respondents after accounting for the matched set (*p* = 0.085 and *p* = 0.098, respectively). Accordingly, a gender-associated difference in these medication-use items is not supported. Variation in the use of medical expulsive therapy remains clinically relevant in selected stone scenarios [15,16], but the present survey does not establish a gender-specific pattern.

### 4.5. Procedural Logistics: Access, Operator, and Technique Patterns

Among procedural-logistics items, coupling-gel use showed a nominal gender-associated difference (*p* = 0.029), with higher reported use among male respondents. JJ-stent placement, ESWL-machine type, access to ESWL, operator, and stone-fragment collection did not differ significantly after accounting for the matched set. These results describe differences in reported procedural practice but do not establish differences in technical competence, caution, or risk aversion. Local protocols, delegation of ESWL operation, training exposure, case selection, and other institutional factors may contribute to the observed variation.

### 4.6. Imaging for Stone-Free Status

Post-treatment imaging for stone-free assessment was broadly similar between female and male respondents in the matched-set-stratified analysis. No evaluated modality reached nominal statistical significance. These survey responses should be interpreted as reported practice preferences rather than direct observation of patient-level follow-up or imaging use [17].

### 4.7. Contraindications and Special Clinical Scenarios

Reported responses concerning SWL-related contraindications and special clinical scenarios were generally similar between female and male respondents. None of the evaluated items reached nominal statistical significance after accounting for the matched set. This similarity indicates that major gender-associated differences were not evident in the contraindication-related items surveyed. However, the survey does not permit inference regarding individual knowledge, decision quality, or actual patient-level management. Radiolucent stones, which had appeared different in the original unadjusted analysis, were not significantly different in the matched-set-stratified analysis (5.8 vs. 5.2; *p* = 0.39). This change illustrates the importance of accounting for the matched design and reinforces the need to avoid interpreting nominal patterns as differences in awareness or competence.

### 4.8. Training and Case Volume as Contextual Factors

Training, procedural exposure, and case volume remain important contextual factors when interpreting gender-associated differences in reported practice. The original unmatched sample contained substantial career-stage differences, which prompted exact matching on career stage to reach the primary analysis set. Training during residency showed a nominal association with gender in the matched-set-stratified categorical analysis (*p* = 0.027), while the overall training-category distribution did not reach nominal significance (*p* = 0.074). These findings are exploratory and do not establish differential training quality. Residual differences in fellowship exposure, institutional resources, procedural autonomy, and other unmeasured aspects of training may remain, as seen in other studies [11,12,18].

### 4.9. Interpretation in a Multinational ESWL Survey Context

The broader ESWL literature shows substantial variation in training, technology, procedural volume, and local practice patterns [19]. The present multinational SIU survey should be interpreted within that context. Because participation was voluntary and geographic response rates were uneven, the findings cannot be assumed to represent all urologists worldwide. Observed gender-associated differences may also reflect regional, institutional, and training-related factors correlated with respondent gender. These possibilities are important contextual considerations; however, causality cannot be established from the present cross-sectional survey.

### 4.10. Comparison with Oncologic Urological Care

These findings can also be considered alongside a parallel international survey from our group examining gender-associated practice patterns in non-muscle-invasive bladder cancer (NMIBC) [20]. That study reported limited gender-associated differences in several guideline-driven NMIBC practices despite differences in procedural volume. The contrast is hypothesis-generating and may reflect differences in clinical context, standardization, respondent mix, or other unmeasured factors. The present data do not establish that clinical stakes or guideline strength cause gender-associated variation to differ between urological domains.

### 4.11. Clinical Implications

The results of our analysis suggest that gender-associated variation in this survey was concentrated in a limited number of reported practices and procedural-volume measures rather than being pervasive across ESWL care. This pattern may help identify specific domains for future investigation, training, and standardization. However, the survey does not establish that the practice pattern of one gender is safer, more effective, or more guideline-adherent than that of the other. Patient-level studies linking clinician characteristics, treatment decisions, and clinical outcomes would be required to evaluate the clinical consequences of these reported differences.

### 4.12. Strengths and Limitations

The strengths of this study include the large multinational sample, inclusion of respondents from 108 countries, administration in seven languages, and detailed assessment of multiple domains of ESWL practice. Important limitations should also be acknowledged. The study was cross-sectional and based on voluntary, self-reported survey responses rather than observed clinical practice or patient records, creating the potential for selection, recall, reporting, and social-desirability bias. The SIU-based respondent sample was not probability-based and should not be considered representative of the global urologist population. Geographic response bias is also possible.

Although the propensity-score matching improved comparability in age, years of clinical practice, continent, and career stage, residual confounding by institutional setting, training exposure, procedural volume, local protocols, and other unmeasured factors may remain. The scored ESWL items were available for 851 matched respondents. While most non-completion was attributable to lack of an ESWL device or zero ESWL volume, selection into the scored-item subgroup remains possible. Because a large number of exploratory comparisons were performed without adjustment for multiple testing, nominally significant findings may include false-positive associations and should be interpreted cautiously. Finally, the individual 0–10 survey items were not treated as validated measures of clinical competence or as a validated composite scale.

### 4.13. Future Directions

This study underscores the need for:

1. Standardized ESWL training curricula, ensuring equity across genders.
2. Increased female representation in endourology and ESWL research [11,12,21].
3. Further studies linking practice patterns to clinical outcomes.
4. Evaluation of gender-based differences across other stone management modalities (URS, PCNL).

Understanding how provider-level characteristics influence procedural decision-making is increasingly important in the era of evidence-based, patient-centered care.

## 5. Conclusions

In this multinational survey of SIU members, most self-reported ESWL practice patterns were similar between female and male respondents after propensity-score matching and matched-set-adjusted inference. Gender-associated differences were observed in several procedural-volume distributions and in a limited number of ESWL-specific items, including coupling-gel use, urine-culture assessment, complete blood count, and repeat SWL within 7 days. Because the study was cross-sectional, voluntary, exploratory, and based on self-report, these associations should not be interpreted as causal effects of gender or as evidence that one gender provides superior or more guideline-concordant care. Rather, the findings identify specific areas of practice variation that warrant confirmation in studies incorporating patient-level clinical context and outcomes.

## Data Availability

The raw data supporting the conclusions of this article will be made available by the authors on request.

## Abbreviations

The following abbreviations are used in this manuscript:

SIU	Société Internationale d’Urologie
ESWL	Extracorporeal Shock-Wave Lithotripsy
URS	Ureterorenoscopy
PCNL	Percutaneous Nephrolithotomy
CT	Computed Tomography
X-KUB	Kidneys, Ureters, and Bladder X-ray
MRI	Magnetic Resonance Imaging
JJ stent	Double-J Ureteral Stent
SFS	Stone-Free Status
SFR	Stone-Free Rate
IRB	Institutional Review Board
SAS	Statistical Analysis System
PSMATCH	Propensity-Score Matching
NMIBC	Non-Muscle-Invasive Bladder Cancer
